# Correlation of DRD2 mRNA expression levels with deficit syndrome severity in chronic schizophrenia patients receiving clozapine treatment

**DOI:** 10.18632/oncotarget.21230

**Published:** 2017-09-23

**Authors:** Liang Liu, Yin Luo, Guofu Zhang, Chunhui Jin, Zhenhe Zhou, Zaohuo Cheng, Guozhen Yuan

**Affiliations:** ^1^ Wuxi Mental Health Center, Nanjing Medical University, Wuxi, China; ^2^ Wuxi Tongren International Rehabilitation Hospital, Nanjing Medical University, Wuxi, China

**Keywords:** chronic schizophrenia, deficit syndrome, mRNA expression phosphoinositide-3 kinase, dopamine D2 receptor, protein kinase B

## Abstract

Schizophrenia is a complex, severe, chronic psychiatric disorder, and the associated deficit syndrome is widely regarded as an important clinical aspect of schizophrenia. This study analyzed the relationship of deficit syndrome severity with the mRNA levels of members of signaling pathways that associate with the pathophysiology of schizophrenia, including the dopamine D2 receptor (DRD2), protein kinase B (AKT1), and phosphoinositide-3 kinase (PI3KCB), in peripheral blood leukocytes (PBLs) of 20 healthy controls and 19 chronic schizophrenia patients with long-term clozapine treatment. The DRD2 expression levels in chronic schizophrenia group were statistically higher than those in controls (t=2.168, p=0.037). Moreover, in chronic schizophrenia group, correlations were observed between the expression levels of DRD2 and PI3KCB (r=0.771, p<0.001), DRD2 and AKT1 (r=0.592, p=0.008), and PI3KCB and AKT1 (r=0.562, p=0.012) and between the DRD2 mRNA levels and the Proxy for the Deficit Syndrome score (r=0.511, p=0.025). In control group, the correlation between PI3KCB expression levels and DRD2 expression levels was only observed (r=0.782, p<0.001). In conclusion, a correlation was observed between increased deficit syndrome severity and elevated expression levels of DRD2 in PBLs of chronic schizophrenia patients receiving long-term clozapine treatment.

## INTRODUCTION

Schizophrenia is a complex, severe, chronic psychiatric disorder with a heterogeneous clinical phenotype [1]. The prevalence of schizophrenia is approximately 1.1% of the population over the age of 18, and 25 million people worldwide are currently affected by this disorder [2]. However, at present, schizophrenia is primarily diagnosed using criterion-based approaches, such as the criteria from the International Classification of Diseases, Tenth Edition (ICD-10), and the Diagnostic and Statistical Manual of Mental Disorders, Fifth Edition (DSM-V) [3]. Biomarkers for the diagnosis, prognosis or therapeutic efficacy of schizophrenia are currently being examined extensively.

Many efforts have been made to investigate the etiology of this disease, including studies focused on genetics, early environmental factors, psychology and neurobiology [4–7]. Gene–environmental interactions have been found to play a crucial role in the development of schizophrenia [8, 9]. Considering these various factors, the development of genomics and molecular biology improved the understanding of the molecular pathophysiology of schizophrenia, especially the related neuronal signaling pathways and the influences of antipsychotic drugs on them [10–13].

The phosphoinositide-3 kinase - protein kinase B (PI3K-Akt) pathway is an important downstream intracellular pathway of DRD2, which is associated with the function and development of central nervous system and the pathophysiology of schizophrenia [10–15]. PI3K-Akt pathway is also the intracellular downstream pathway of glutamate, serotonin, dysbindin, disrupted in schizophrenia-1 (DISC-1), and neuregulin 1 (NRG1), which are all the targets for mood stabilizers and antipsychotic drugs [10, 15, 16]. Almost all aspects of the cell developments, such as growth, proliferation, metabolism and apoptosis, were modulated by PI3K-Akt pathway. Reduced PI3K/Akt activity destroys schwann cells and oligodendrocytes, damages axonal guidance and cell–cell interactions, and reduces synaptic number, which may all lead to schizophrenia [16, 17]. Dysregulation of PI3K-AKT signaling cascade could critically disturb the neurodevelopmental process via genetic and environmental risk, and has been regarded as a root cause of several neurodevelopmental diseases, including schizophrenia [18]. The murine model of Akt3 genetic deficiency exhibited selective deficits of temporal order discrimination and spatial memory, tasks critically dependent on intact prefrontal-hippocampal circuitry, which related to schizophrenia [19]. Double deficiency of Akt1 and Nrg1 can result in the impairment of social cognitive functions, which might be pertinent to the pathogenesis of schizophrenia-related social cognition which related to schizophrenia [20].

According to recent researches, DRD2-PI3K-AKT pathway could have at least three intracellular downstream segments, which play important roles in cell growth and proliferation [21, 22]. Firstly, AKT could induce the inactivation of forkhead family of transcriptional regulators (FOXOs) by promoting the phosphorylation of them. And FOXOs could contribute to neural stem cell renewal and proliferation [23–25] and also have prominent roles in the development of neuronal circuits [26]. Secondly, AKT could also induce mammalian target of rapamycin (mTOR) to be phosphorylated. mTORs could regulate intracellular protein synthesis, synaptic plasticity, neuronal morphology, and the pathophysiology of schizophrenia [27]. Thirdly, a multifunctional serine/threonine kinase, glycogen synthase kinase-3 (GSK-3) also could be inactivated by AKT. GSK-3 can regulate a series of neuron response, such as microtubule dynamics [28].

Moreover, Schizophrenia is a complex disorder characterized by a high degree of variability in its performance of negative, positive, and cognitive symptoms. The presentation of negative symptoms (i.e., blunted affect, lack of will, impaired social function) and cognitive impairment (i.e., impaired working memory and executive function) are the core and longest lasting symptoms of schizophrenia [29–32]. In particular, the deficit syndrome of schizophrenia is defined as the stable persistence of two or more negative symptoms (i.e., alogia, affective flattening, lack of focus, loss of interest in social activities, loss of interest or reduced emotional range) for at least one year [33, 34]. The deficit syndrome of schizophrenia associated with poorer response to therapy and severer disease course [35]. The arisement of the definition of deficit syndrome subtype was an attempt to increase the clinical homogeneity of schizophrenia. The Proxy for the Deficit Syndrome (PDS) was the most common method to evaluate the deficit syndrome of schizophrenia based on the following calculation of Positive and Negative Syndrome Scale (PANSS) [36] items: affective flattening (N1) + lack of spontaneity and fluency in conversation (N6) - (hostility (P7) + guilt (G3) + anxiety (G2) + depression (G6)) [3, 37]. The present study aimed to explore the gene expression patterns of DRD2, AKT1, and PI3KCB in peripheral blood lymphocytes (PBLs) of chronic schizophrenia patients with deficit syndrome.

## RESULTS

### Comparisons of the DRD2, AKT1, and PI3KCB expression levels between the two groups

**Figure 1:**
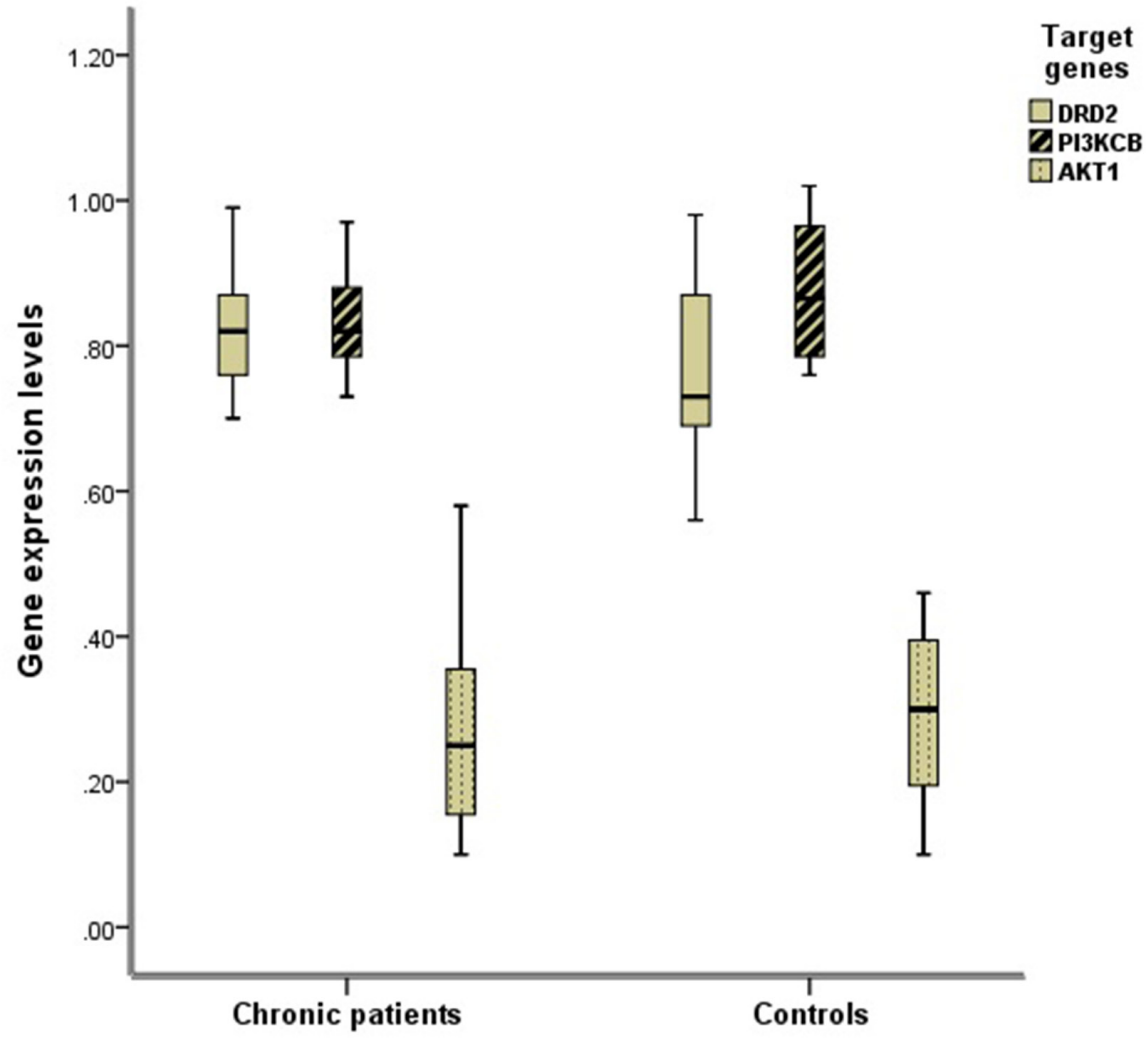
Comparison of the expression levels of DRD2, AKT1, and PI3KCB between two groups The mRNA expression levels of DRD2, AKT1, and PI3KCB in PBLs were normalized respectively by the ratio of their CT values and that of GAPDH as internal housekeeping gene. The DRD2 expression levels in chronic schizophrenia group were statistically higher than those in controls (t=2.168, p=0.037).

The DRD2 expression levels in chronic schizophrenia patients were statistically higher than those in controls (t=2.168, p=0.037). The PI3KCB (t=1.469, p=0.142) and AKT1 (t=0.500, p=0.620) mRNA levels demonstrated no significant differences between chronic schizophrenia patients and healthy controls (Table 1, Figure 1).

**Table 1:** Comparison of the expression levels of DRD2, AKT1 and PI3KCB between two groups (means±S.D.)

	Chronic schizophrenia patients	Controls	t	*p*
	(n=19)	(n=20)		
DRD2	0.83±0.09	0.76±0.11	2.168	0.037*
AKT1	0.27±0.13	0.29±0.12	0.500	0.620
PI3KCB	0.83±0.08	0.87±0.09	1.469	0.142

The mRNA expression levels of DRD2, AKT1, and PI3KCB in PBLs were normalized respectively by the ratio of their CT values and that of GAPDH as internal housekeeping gene. The independent-samples T test was used. * p<0.05.

### Correlations between DRD2, AKT1, and PI3KCB expression levels in two groups

**Figure 2:**
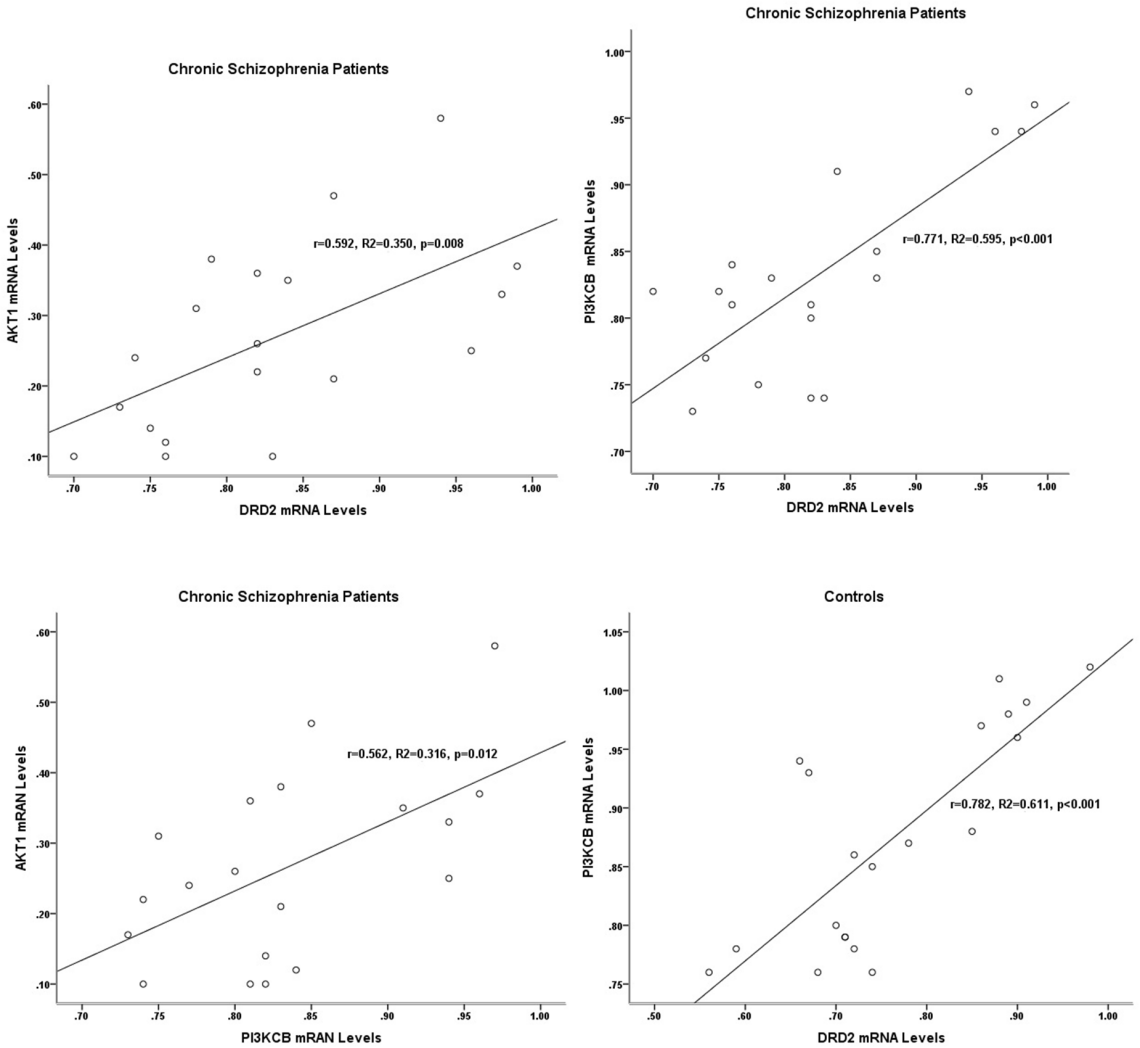
Correlations between the gene expression levels of DRD2, AKT1, and PI3KCB in two groups In chronic schizophrenia patients, significant correlations were detected between the expression levels of DRD2, AKT1, and PI3KCB. In controls, the significant correlation was only found between the expression levels of PI3KCB and DRD2.

In chronic schizophrenia group, there were statistical correlations between the expression levels of PI3KCB and DRD2 (r=0.771, p<0.001), DRD2 and AKT1 (r=0.592, p=0.008), and PI3KCB and AKT1 (r=0.562, p=0.012). And only the correlation of PI3KCB and DRD2 expression levels was significantly detected in the control group (r=0.782, p<0.001) (Table 2, Figure 2).

**Table 2:** Correlations between the mRNA levels of DRD2, AKT1, and PI3KCB (R-values)

	Chronic schizophrenia patients			Controls		
	DRD2	AKT1	PI3KCB	DRD2	AKT1	PI3KCB
DRD2	1.000	0.592**	0.771**	1.000	0.366	0.782**
AKT1	0.592**	1.000	0.562*	0.366	1.000	0.274
PI3KCB	0.771**	0.562*	1.000	0.782**	0.274	1.000

* p<0.05, ** p<0.01.

### Relationships of DRD2, AKT1, and PI3KCB expression levels with PANSS scores of chronic schizophrenia group

**Figure 3:**
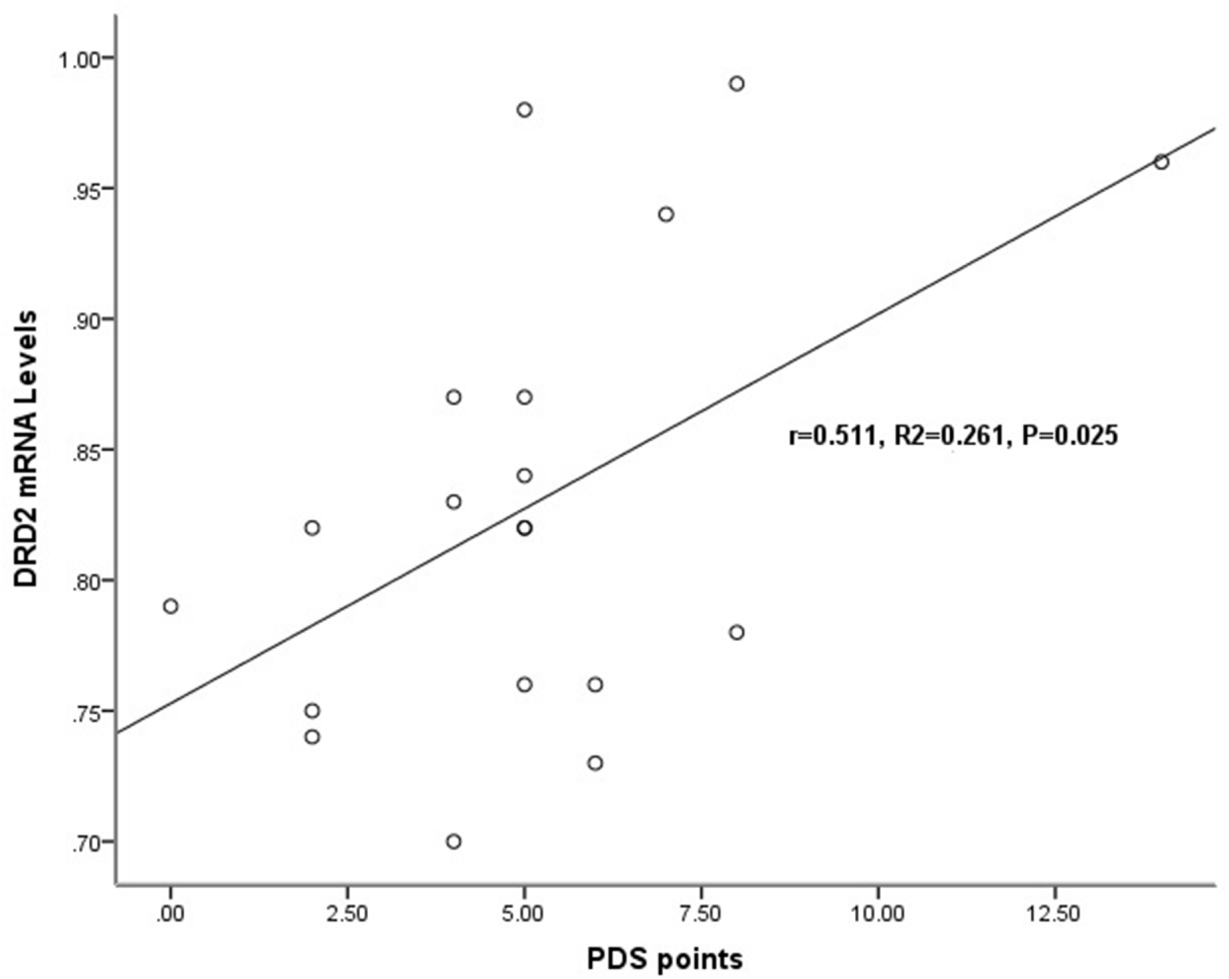
Relationship of the DRD2 mRNA levels with the severity of deficit syndrome in chronic schizophrenia patients In chronic schizophrenia patients, a significant relationship was detected only between the mRNA expression levels of DRD2 (r=0.511, p=0.025) and the Proxy for the Deficit Syndrome (PDS) score.

In chronic schizophrenia group, there was no significant relationship between target genes expression levels and the total PANSS score, the positive symptom score, the negative symptom score or the general pathological symptom score. However, the relationship of DRD2 mRNA levels (r=0.511, p=0.025) and the PDS score was significantly observed (Table 3, Figure 3).

**Table 3:** Relationships between the expression levels of DRD2, AKT1, and PI3KCB and the PANSS scores of chronic schizophrenia patients (R-values)

	DRD2	PI3KCB	AKT1
Total PANSS score	0.243	-0.022	0.037
Positive symptom score	0.240	0.249	0.191
Negative symptom score	-0.387	-0.158	-0.143
General pathological symptom score	-0.153	-0.128	-0.109
Proxy for the Deficit Syndrome score	0.511*	0.456	0.150

Spearman's correlation coefficients are shown. * p<0.05.

## DISCUSSION

Schizophrenia is a complex disorder characterized by a high degree of variability in its performance of negative, positive, and cognitive symptoms. Although with the same diagnosis, schizophrenia patients may display great different symptoms [38]. This variation has complicated genomic and molecular biological studies of schizophrenia. Negative symptoms and cognitive deficits are the most persistent manifestations of schizophrenia and the core symptoms of this disease [29–32]. However, chronic schizophrenia patients exhibit homogeneous disease characteristics, focusing on chronic schizophrenia would generate influential and helpful insights for future research concerning the pathophysiology of this disease. And the development of genomics and molecular biology improved the understanding of the molecular pathophysiology of schizophrenia, especially the DRD2-PI3K-Akt signaling pathways and the influences of antipsychotic drugs on them [10–13]. The present study analyzed the gene expression patterns of DRD2, AKT1, and PI3KCB in peripheral blood lymphocytes (PBLs) of chronic schizophrenia patients with deficit syndrome.

We found that the mRNA levels of DRD2 were significantly higher in chronic schizophrenia patients than those in controls, which is consistent with the findings of three studies. For instance, the increased DRD2mRNA expression levels have also been found by Zvara et al. in PBMCs of drug-naïve or drug-free schizophrenia patients with a varied duration of illness and severe symptoms [39], by Brito-Melo et al. in T cell subsets (CD4 and CD8) of medicated chronic schizophrenia patients with duration of illness >10 years [40], and by Kordi-Tamandani et al. in whole blood samples of schizophrenia patients, not mentioned the medical treatment [41]. However, there have been some inconsistent reports in the literature. Ahmadian et al., Cui et al., Yao et al. and our laboratory have found no significant difference in DRD2 mRNA expression levels in peripheral blood of schizophrenia patients [42–45]. We have analyzed PANSS scores in the different patient groups of schizophrenia in a previous paper [45]. And the PANSS scores of chronic schizophrenia patients this time (76.16±9.22) were statistically higher (t=2.0685, P=0.0445) than chronic schizophrenia patients in the previous research (70.32±9.57).

We also found that the PI3KCB and AKT1 mRNA levels demonstrated no significant differences between chronic schizophrenia patients and controls. About PI3KCB mRNA levels in peripheral blood samples of schizophrenia patients, there was only our previous one paper reported that the PI3KCB mRNA expression levels in the acute schizophrenia patients were significantly lower than those in the healthy controls [46], and this time in the chronic schizophrenia patients with long-term clozapine treatment, PI3KCB mRNA expression levels demonstrated no significant change. In the same previous paper, we have found the AKT1 mRNA levels in peripheral blood samples of acute schizophrenia patients were significantly higher than those in the healthy controls [46], just like the researches in peripheral blood mononuclear cells (PBMCs) of Kumarasinghe et al. [47] and Xu et al. [48]. And this time, we reported that AKT1 mRNA levels demonstrated no significant differences between chronic schizophrenia patients with long-term clozapine treatment and controls. Kumarasinghe et al. also found that AKT1 gene expression levels returned to control levels after 6 weeks treatment with risperidone or risperidone in combination with haloperidol [47]. But Xu et al. reported AKT1 expression levels have no change after treatment with oral second generation or atypical antipsychotics (SGA) [48]. There are, of course, some opposite findings. Noto et al. found no significant difference in AKT1 mRNA expression level in whole blood samples between 174 antipsychotic naïve first episode psychosis and 77 healthy controls [49]. van Beveren et al. reported that significantly decreased PBMC expression of AKT1 in all the antipsychotic-free or -naive patients, florid psychotic and remitted patients [50].

These inconsistent results of mRNA expression levels of DRD2, PI3KCB and AKT1 may have been obtained because in contrast to the above studies, firstly, our samples were collected from chronic schizophrenia patients receiving long-term treatment with single clozapine. The deficit syndromes of chronic schizophrenia patients and the pharmacological effects of clozapine might have influenced the examined mRNA levels. Secondly, the expression level of DRD2, PI3KCB, and AKT1 in peripheral blood may not an index of schizophrenia, but only an index of psychiatric symptoms.

With regard to the correlations between the mRNA levels of DRD2, AKT1, and PI3KCB, significant correlations were detected between the mRNA levels of AKT1 and DRD2, AKT1 and PI3KCB, DRD2 and PI3KCB in chronic schizophrenia patients. In healthy controls, the only statistical correlation was observed between the mRNA levels of PI3KCB and DRD2. PI3K-Akt pathway was the non-classical downstream signaling pathway of DRD2. And the combination of DA and DRD2 receptor leads serially to the activation of PI3K and the dephosphorylation and inactivation of AKT [14, 15]. In chronic schizophrenia patients receiving long-term clozapine treatment, the pharmacological effects of clozapine might influence the functional status of the DRD2-PI3K-Akt signaling cascade. Studies have shown the superiority of clozapine to typical agents for treating the negative symptoms and positive symptoms of schizophrenia [51]. The time lag between the start of treatment and the therapeutic effect of antipsychotic agents suggests that changes in gene expression contribute to their efficacy [52–55].

In chronic schizophrenia group, there was a significant relationship detected exclusively between DRD2 gene expression levels and the PDS score, not between the expression levels of any examined gene and the total PANSS score, the positive symptom score, the negative symptom score or the general pathological symptom score. The proposition of deficit syndrome was aiming for reducing the clinical heterogeneity of schizophrenia [33, 56]. The deficit syndrome is featured with persistent and primary negative symptoms which stay through clinical decompensation and stability stage. Some researches have reported the deficit syndrome could help to differentiate the risk factors, clinical features and prognosis, pharmacological response profiles, neurocognitive and biological of schizophrenia patients. And the dysfunction of neural circuitry in dorsolateral prefrontal cortex (DLPFC) might be the pathological basis of deficit syndrome [35, 56]. The dopamine hypothesis of schizophrenia also suggests that dysfunction of the neural circuitry, such as subcortical hyperdopaminergia and prefrontal hypodopaminergia [57]. Our results showed that DRD2 gene expression levels in PBLs were correlated with the deficit syndrome of chronic schizophrenia patients. But the researches on the relation between DRD2-PI3K-AKT pathway and the deficit syndrome of schizophrenia were relatively scarce. Previous studies have found that deficit schizophrenia patients have decreased plasma levels of high oxalate (dopamine metabolites, PHVA) levels, and increased plasma levels of 3-methyl-4-hydroxy phenyl ethylene glycol (Norepinephrine metabolites, MHPG), and the symptoms of deficiency were related to the increase of NE level and the decrease of DA level [58]. Some papers reported that more homozygous Val/Val in catechol-O-methyltransferase (COMT) Val^158^Met genotypes were found (31 vs. 17%) in the non-deficit schizophrenia patients compared to the deficit schizophrenia subgroup [59], and SNP8NRG241930 in NRG1 were associated with non-deficit schizophrenia [60]. COMT and NRG1 are both the upstream signals of DRD2-PI3K-AKT pathway. So, the DRD2-PI3K-AKT pathway was supposed to involve in neuropathogenesis of deficit syndrome of schizophrenia.

This research also had some limitations. Firstly, the small sample size was the major limitation of this study, which may have led to insufficient statistical power to detect some slight difference. Secondly, the chronic schizophrenia patients were monotherapied with clozapine for more than 6 months before recuited in the study, but we did not limit the antipsychotic drugs which might be used before that. So, the possible long-term effects of antipsychotic drugs ever used were not considered. Thirdly, there are evident limitations of using peripheral blood to explore a central nervous system disease, but researches of postmortem brain samples circumvent the corresponding confounding factors, such as effects of long-term hospitalization and lifetime exposure to antipsychotic drugs [61, 62]. The researches of peripheral mRNA expression could provide a chance to understand the gene expression profile of central nervous system because, the gene expression levels of many kinds of biologically substances display the similar gene expression patterns in peripheral blood and prefrontal cortical tissue [50, 61, 62].

In conclusion, a correlation was observed between increased deficit syndrome severity and elevated DRD2 expression levels in PBLs of chronic schizophrenia patients receiving long-term clozapine treatment. However, considering the limited *in vivo* evidence concerning the proteins phosphorylation levels which related to the functional status of PI3K-Akt pathway and the understanding that PI3K-Akt pathway is activated by several different regulatory mechanisms to exert its biological effects [63], the alterations in the DRD2-PI3K-Akt pathway have not been fully elucidated as biological markers of schizophrenia.

## MATERIALS AND METHODS

### Participants

From July 2011 to September 2012, 19 chronic schizophrenia patients were recruited from clinical psychiatry department, Wuxi Mental Health Center, Nanjing Medical University; and 20 healthy controls were recruited from physical examination center of Wuxi Tongren International Rehabilitation Hospital. Each chronic schizophrenia patient received the routine examination and psychiatric examination, and all completed the criteria of schizophrenia according to the DSM-IV-TR [64]. The courses of disease of chronic schizophrenia patients were all longer than 2 years [65], and had been treated with clozapine monotherapy for at least 6 months (drug dose fulfilled the treatment dose). And their symptom features accorded with the criteria for deteriorated schizophrenia subtype or residual schizophrenia subtype of Chinese Classification and Diagnostic Criteria of Mental Disorders, Third Version (CCMD-3) [65] and residual schizophrenia subtype of the DSM-IV-TR [64].

All subjects were Han Chinese, who had no previous history of drug abuse, nervous system disease and immune disease. The health controls group had no history in neuropsychology. The female subjects were not in lactating, menstruating or pregnant stage when recruited. There was no significant difference in gender and age between two groups. Their demographic and clinical characteristics are shown in Table 4.

**Table 4:** Demographic and clinical characteristics of the subjects

	Chronic schizophrenia patients	Controls	Statistic
	(n=19)	(n=20)	
Mean age (S.D.)	44.42 (5.50)	39.80 (8.71)	t=1.968, p=0.057
Gender (male) (%)	11 (57.89)	9 (45.00)	χ^2^=0.648, p=0.421
Duration of disease (months) (S.D.)	>24	NA	NA
Mean total PANSS score (S.D.)	76.16(9.22)	NA	NA
Age of onset (S.D.)	25.47(9.96)	NA	NA
Duration of therapy (months) (S.D.)	20.41(7.82)	NA	NA
Number of incidence (S.D.)	8.16(2.14)	NA	NA

The variance of the age in the two groups was equivalent (F=2.508, p=0.057).

The psychotic symptoms of the patients were quantified with PANSS by one rater just in the same day when the blood samples were drawn. Then the deficit syndrome of schizophrenia was evaluated by the PDS calculated with PANSS items which was mentioned previously in the introduction.

### Ethical considerations

The research is based on the principles described in the Helsinki declaration. The research was approved by the ethics committee of the Wuxi mental health center. Before the research, all the participants were informed the procedures and corresponding rights. And every subject or major legal guardian, in case the decision-making ability of participant was limited, had signed the informed consents.

### Methods

### Sample collection and RNA isolation

Each subject was drawn 2 ml of fasting ulnar vein blood in vacutainer tube with heparin. Total ribonucleic acid (RNA) of each sample was extracted with QIAamp RNA Blood Mini Kit (Qiagen Co., Germany) contained DNase processing steps. The absorbance of RNA samples were examined to conduct the quality check using Bioanalyzer 2100 System, Agilent. The RNA integrity numbers (RIN) of RNA samples were 8.21±0.36, and the 28S/18S ratios were 1.88±0.45, which all displayed the good integrity.

RNA extraction and reverse transcription were conducted in 3 hours after drawing blood samples in order to reduce the RNA degradation. To minimize batch effects, the fasting blood sample was consistently drawn at 6:00 am, and RNA extraction and reverse transcription were performed by the same laboratory technician utilizing identical experimental procedures.

### Real-time polymerase chain reaction (PCR) and relative quantitative analysis

Total RNA was used as template to synthesize the first-strand cDNA with QuantiTect Reverse Transcription Kit (Qiagen Co., Germany). Each reaction mixture had 400 ng of RNA template, and was placed in a water bath at 42 °C for a period of 20 minutes, and then in a water bath at 95 °C for a period of 3 minutes to terminate the reaction. Finally, the reaction production was stored at -80 °C.

To conduct real-time PCR reaction, QuantiFast Probe PCR Kit (Qiagen Co., Germany) and QuantiFast Probe Assays (Qiagen Co., Germany) were used in ABI Prism 7500 PCR instrument (ABI Co., USA). The probe assays were prefabricated with probes and primers for glyceraldehyde-3-phosphate dehydrogenase (GAPDH) as internal control, including probes and primers of Hs_AKT1P_QF_1 assay (QF00525315) for AKT1, Hs_PI3KCB_QF_1 assay (QF00069097) for PI3KCB, and Hs_DRD2_QF_1 assay (QF00103047) for DRD2. The volume of each reaction system was up to 25 μl with the reaction conditions as pre-denaturing at 95°C for 5 min, then 40 cycles of denaturing at 95°C for 30 s and annealing at 60°C for 30 s. Each sample was conducted 3 parallel reactions in order to calculate the average CT value.

**Figure 4:**
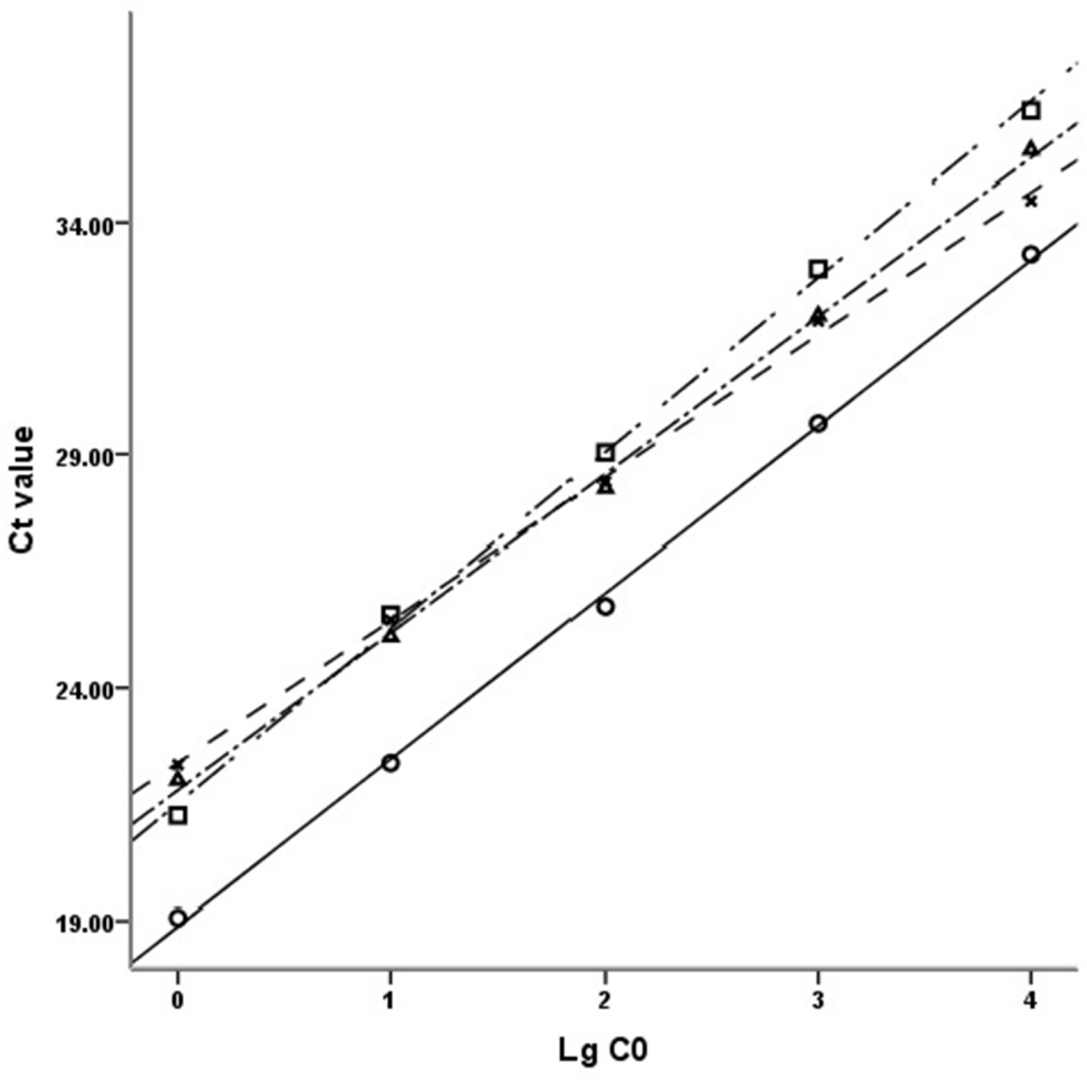
Standard curves of DRD2, GAPDH, Akt1 and PI3KCB The standard curves of DRD2, GAPDH, Akt1 and PI3KCB with 10-fold gradient dilution displayed good linear relationship. Ct value: Cycle threshold value; LgC0: Logarithm of the initial concentration; □: DRD2, y=-3.7679x + 40.352, R^2^=0.9983; ○: GAPDH, y=-3.5773x + 36.767, R^2^=0.9989; ∆: AKT1, y=-3.3937x + 38.760, R^2^=0.9984; ×: PI3KCB, y=-3.0661x + 37.709, R^2^=0.9986.

The double standard curve method [66] was chosen to obtain the relative quantities of each target gene (Figure 4). Using the data from each amplification reaction plate, standard curves for internal control gene and target genes were figured out with the same threshold. So, the original mRNA levels of target genes and GAPDH were figured out by their respective standard curves and CT values. Then, the relative mRNA levels of target genes were normalized by the ratio of CT values of target gene and that of GAPDH respectively, and this normalized process avoids the discrepancies in amplification results which resulting from the differences of the mRNA amount initially added in the reaction system. This double standard curve method makes the accurate copy number or concentration criterion not necessary and the preparation of standard curves relatively simpler.

### Statistical analyses

Statistical software (SPSS version 19) was used for statistical analysis. The homogeneity test of variances of the target genes expression levels in both groups were conducted firstly. Then, to compare the differences of expression levels of target genes between the two groups, Independent-samples T test was chosen. And Pearson’s correlation analysis for analyzing the correlations between the target genes expression levels, and Spearman correlation analysis for analyzing the relationships between each target gene expression level and PANSS scores were chosen. All data were displayed in terms of means ± standard deviation (S.D.) with a 2-sided p value ≤0.05 as statistical significance.

## Abbreviations

DRD2: dopamine D2 receptor
AKT: protein kinase B
PI3K: phosphoinositide-3 kinase
PBLs: peripheral blood leukocytes
NRG1: neuregulin 1
GSK-3: glycogen synthase kinase-3
DISC-1: disrupted in schizophrenia-1
FOXOs: forkhead family of transcriptional regulators
mTOR: mammalian target of rapamycin
CCMD-3: Chinese Classification and Diagnostic Criteria of Mental Disorders, Third Version
ICD-10: International Classification of Diseases, Tenth Edition
DSM-IV-TR: Diagnostic and Statistical Manual of Mental Disorders, Fourth Edition, Text Revision
PANSS: Positive and Negative Syndrome Scale
PDS: Proxy for the Deficit Syndrome
